# Supplementary material for: Synthesis and Characterization of Phase-Separated Extracellular Condensates in Interactions with Cells
Source: bioRxiv. 2025 Mar 26:2025.03.24.644961. Preprint. [Version 1] doi: 10.1101/2025.03.24.644961 (PMC11974749; doi:10.1101/2025.03.24.644961)
Supplement: Supplement 1 [file media-1.pdf]

Supplementary Information to:

Synthesis and Characterization of Phase-Separated Extracellular Condensates in Interactions with Cells

Aida Naghilou[1,2], Tom M.J. Evers[1,2], Oskar Armbruster[3], Vahid Satarifard[2], Alireza Mashaghi[1,2]*

[1] Medical Systems Biophysics and Bioengineering, Leiden Academic Centre for Drug Research, Faculty of Science, Leiden University, 2333CC, Leiden, The Netherlands

[2] Laboratory for Interdisciplinary Medical Innovations, Centre for Interdisciplinary Genome Research, 2333CC, Leiden University, Leiden, The Netherlands

[3] Institute of Synthetic Bioarchitectures, Department of Biotechnology and Food Science, BOKU University, 1190 Vienna, Austria

*Correspondence: a.mashaghi.tabari@lacdr.leidenuniv.nl

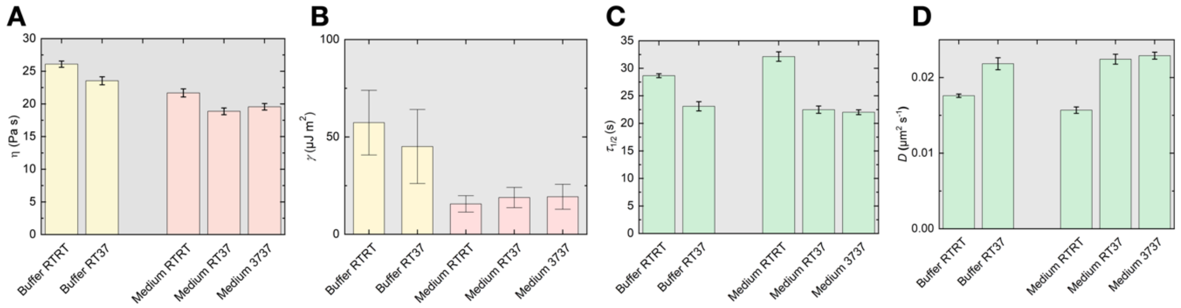

Supplementary Figure S 1: Comparison of the material properties of pK-HS condensates in various conditions. A) Viscosity (η) measured as well as **B)** Surface energy density (γ) measured with SPM **C)** half-time of recovery ($\tau_{1/2}$) and **D)** diffusion coefficient (D) measured with FRAP. **Buffer RTRT:** pK-HS condensates formed in 0.15 M KCl buffer and RT, measured at RT. **Buffer RT37:** pK-HS condensates formed in 0.15 M KCl buffer and RT, measured at 37°C. **Medium RTRT:** pK-HS condensates formed in culture medium and RT, measured at RT. **Medium RT37:** pK-HS condensates formed in culture medium and RT, measured at 37°C. **Medium RTRT:** pK-HS condensates formed in culture medium and 37°C, measured at 37°C.

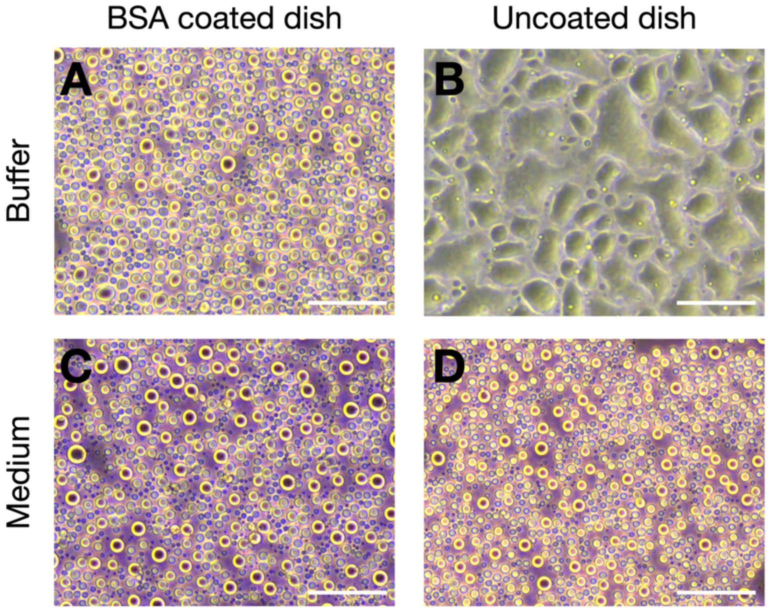

Supplementary Figure S 2: Comparison of the morphologies of pK-Hs condensates depending on surface passivation and medium. A) Condensates formed in 0.15 M KCl buffer on BSA coated dish **B)** Condensates formed in 0.15 M KCl buffer on uncoated dish **C)** Condensates formed in culture medium on BSA coated dish **D)** Condensates formed in culture medium on uncoated dish. Scale bar is 50 µm.

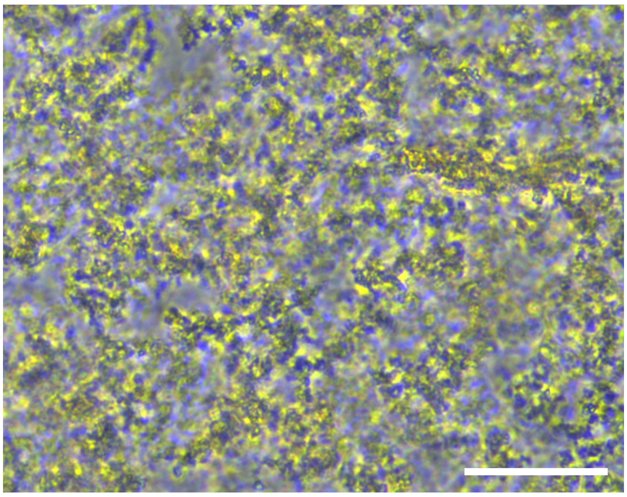

Supplementary Figure S 3: pK-H interactions in culture medium. Scale bar is 50 µm.

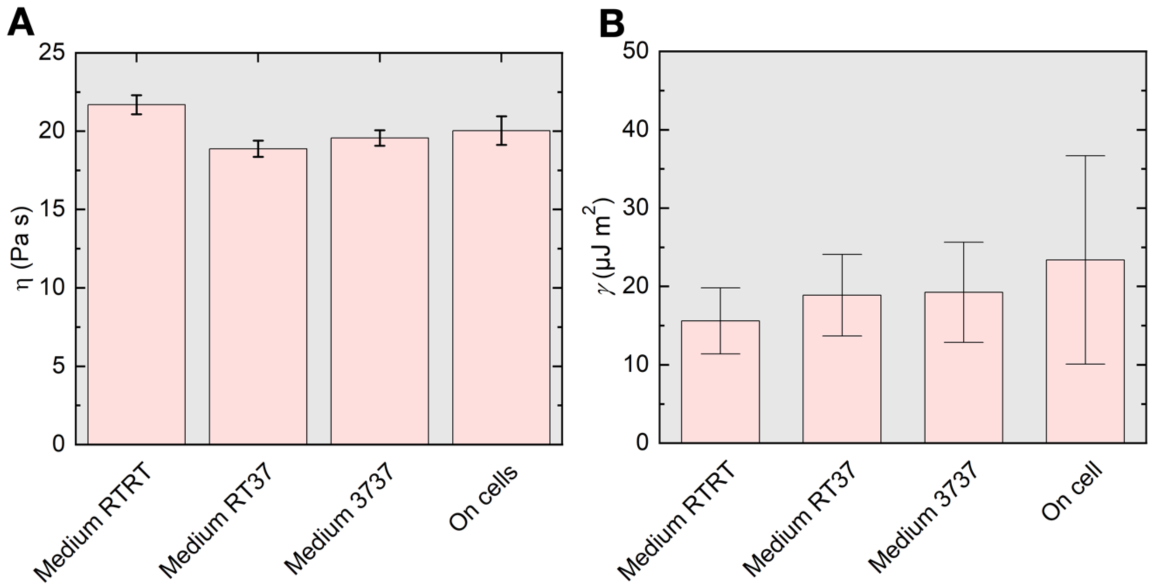

Supplementary Figure S 4: Comparison of the material properties of pK-HS condensates in various conditions and on fibroblast cells. A) Viscosity (η) as well as **B)** Surface energy density (γ)measured with SPM (Eq. 12-15) for **Medium RTRT:** pK-HS condensates formed in culture medium and RT, measured at RT. **Medium RT37:** pK-HS condensates formed in culture medium and RT, measured at 37°C. **Medium RTRT:** pK-HS condensates formed in culture medium and 37°C, measured at 37°C. **On cells:** pK-HS condensates formed in culture medium and 37°C, measured at 37°C while resting on cells.

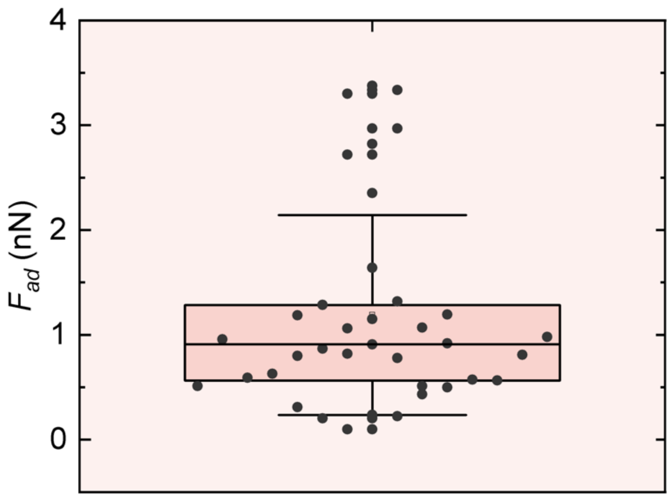

Supplementary Figure S 5: The distribution of the adhesion forces (F_{ad}) between the laminin coated cantilever and pK-HS condensates.
